# Lymphatic Filariasis Presenting as a Swelling over the Upper Arm: A Case Report

**DOI:** 10.5704/MOJ.1803.016

**Published:** 2018-03

**Authors:** GM Sharma, AR Bhardwaj, NR Relwani, S Dubey

**Affiliations:** Department of Orthopaedics, Sir HN Reliance Foundation Hospital, Mumbai, India; ^*^Department of Orthopaedics, Deen Dayal Upadhyay Hospital, New Delhi, India; ^**^Department of Orthopaedics, Mahatma Gandhi Missions Medical College, Kamothe, India; ^***^Department of Orthopaedics, Dr Baba Saheb Ambedkar Hospital, New Delhi, India

**Keywords:** lymphatic filariasis, eosinophilia, diethylcarbamazine

## Abstract

Filariasis continues to be one of the endemic problems worldwide with 40% of the cases in India. We report a case of lymphatic filariasis in a 32-year old female who presented with a non-tender swelling over left upper arm. Blood sample showed no eosinophilia while the FNAC was diagnostic of W. bancrofti. Patient responded well with oral diethylcarbamazine. High index of suspicion of filariasis is indicated when dealing with a swelling of unknown cause especially in filariasis endemic areas.

## Introduction

Filariasis is a vector borne disease especially prevalent in tropical and subtropical regions and caused by slender thread-like filarial worms from the superfamily of Filarioidea, which has an affinity towards skin and subcutaneous tissue or lymphatic system^1^. While the nematode-like Onchocerca volvulus and Loa loa cause filariasis of the skin, Wuchereria bancrofti, Brugia malayi and Brugia timori cause lymphatic filariasis in the descending order^1,2^. Wuchereria bancroftian filariasis produces a wide range of clinical manifestations depending upon the phase and duration. Microfilaremia and paradoxical eosinophilia (lowest count at night) are the striking features in the acute phase. The chronic phase is usually characterized by presence of lymphadenopathy in lower limbs, retroperitoneal tissues, lymphedema, hydrocele and elephantiasis^3^. Microfilaria are also usually absent in early and late phase of the disease. Circulating filarial antigen testing is more sensitive and can detect the presence of the nematode, even in early stages. However, the test has cost limitations^2^.

We report a rare case of Bancroftian filariasis in a 32-year old female presenting with a small subcutaneous swelling at the lateral aspect of the upper arm.

## Case Report

**Fig. 1:**
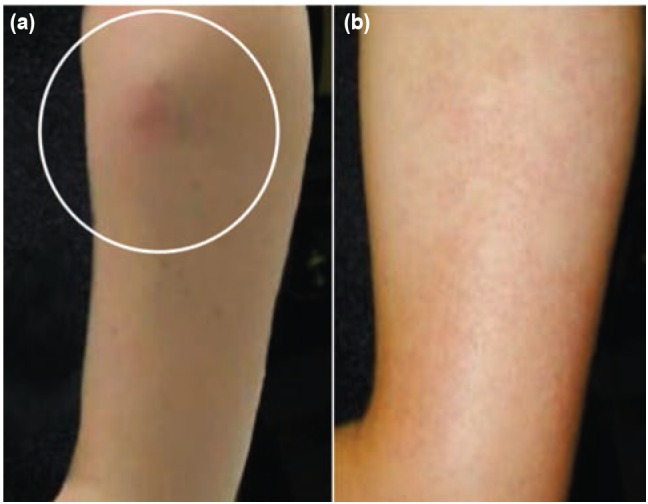
(a) Pre-treatment clinical photograph and (b) Clinical photograph after treatment.

A 32-year old female homemaker presented to the orthopaedic out-patient department with complaints of a painless swelling over the lateral aspect of her left upper arm for five months. There was no antecedent history of injury or trauma. There was no history of similar swelling elsewhere in the body or of constitutional symptoms associated. On examination, there was a 2x2.5cm non-tender, immobile swelling which was firm in consistency and adherent to the skin (Fig. 1a). It was not warm to touch. There were 2-3 lymphnodes, non-tender, in the left axilla. The systemic examination was normal. Haematological investigations were completely normal.

**Fig. 2:**
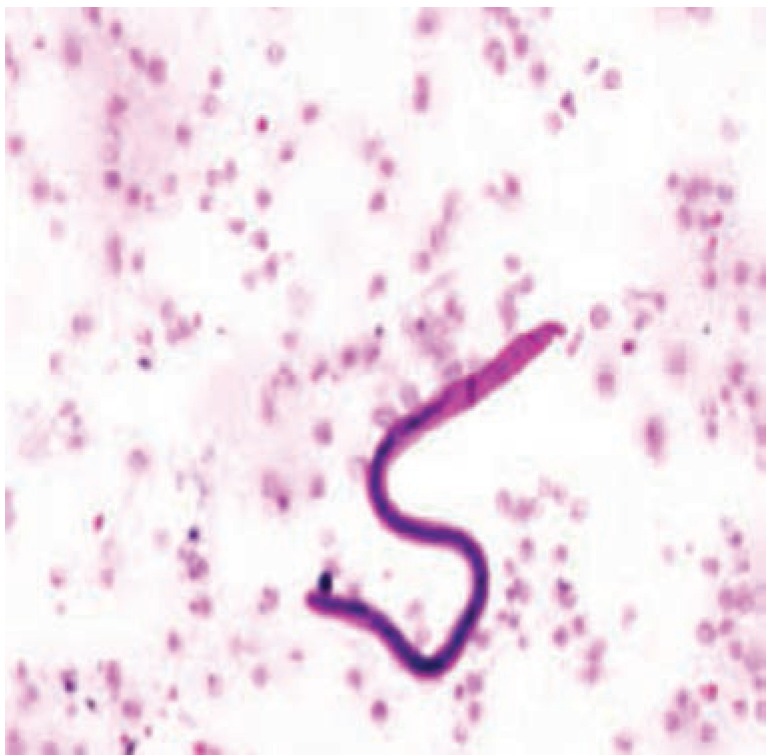
Cytological smear showing W. bancrofti (Giemsa stain with 100X oil magnification).

Fine needle aspiration cytology (FNAC) was done, with aspiration of about 0.3ml of clear fluid. The smear showed presence of thin, slender colourless thread like larvae with blunt head and tail tip free of nuclei were identified which raised the suspicion of W. bancrofti (Fig. 2). After identification of W. bancrofti, a night blood sample failed to show the growth of microfilariae. The patient was started on oral Diethylcarbamazine 100mg thrice a day for 21 days. There was a tremendous decrease in the swelling at the end of four weeks (Fig. 1b). There was disappearance of the axillarylymphadenopathy at the end of three months. The patient was closely followed-up for two years and showed no signs of recurrence.

## Discussion

Filariasis infection is caused by the nematode worms Wuchereria bancrofti, Brugia malayi and Brugia timori transmitted by mosquitoes. Humans are the definitive host for these infections while mosquito acts as an intermediate host. This infection is endemically seen in tropical regions especially India (with the exception of some parts of northern India), China, Indonesia, and parts of Africa^2,3^.

The clinical manifestation can vary according to the phase which can be acute or chronic. Patients in the endemic areas can be asymptomatic for many years. The acute phase is usually associated with microfilaremia and eosinophilia. Microfilaremia is usually detected in blood or skin specimens, but its absence does not rule of the presence of filariasis. In our case, the classical microfilaria of W. bancrofti were seen on cytological examination on peripheral smear which was pathognomic of the disease. Eosinophils can be normal in the majority of cases, like in ours.

Cytological examination with FNAC proves to be an investigation of choice especially in patients with presence of swelling with difficult clinical diagnosis. Microfilariae can be present in breast, thyroid, liver and lungs. Presence of these parasites in the arm as a nodule has been rare and these tests although easily available, lack sensitivity and specificity^4^. Circulating filarial antigen test (CFA) has recently revolutionized the diagnosis of filariasis due to its ease to perform with just a finger prick., non-dependence on microfilariae, avoidance of diurnal variations as opposed to cytological tests and high sensitivity and specificity as compared to the other investigations^3,5^. The major disadvantage of this test is the high cost of the kit, which makes its access difficult for the masses.

The treatment should be multifactorial involving various steps such as vector control in the endemic areas, prompt accurate diagnosis and mass drug administration. The present patient was from an endemic area with a higher prevalence of filariasis, which was initially not suspected and treated symptomatically. The patient responded well to the oral treatment with diethylcarbamazine with swelling regression and no recurrence at two-year follow-up.

The possibility of filariasis should always be kept in mind especially in patients from endemic areas with swelling of unknown cause. Cytological and clinical suspicion together form the mainstay for the diagnosis. Oral treatment with diethylcarbamazine gives excellent results with regression of the infection.

## Conflict of Interest

There was no conflict of interest in this study..
